# Extrahepatic Bile Duct Injury Caused by Blunt Abdomen Trauma: A Case Report

**DOI:** 10.7759/cureus.25662

**Published:** 2022-06-04

**Authors:** Shaurav Ghosh, Farah Naaz Kazi, J V Pranav Sharma

**Affiliations:** 1 General Surgery, Vydehi Institute of Medical Sciences and Research Centre, Bangalore, IND; 2 Surgery, Vydehi Institute of Medical Sciences and Research Centre, Bangalore, IND

**Keywords:** bile, subhepatic space, extrahepatic bile duct injury, abdomen trauma, blunt trauma

## Abstract

Traumatic injuries to the extra-hepatic biliary tract are uncommon and may be suspected intraoperatively by the presence of bile-stained fluid in the subhepatic area. We present a case of injury to the common bile duct in a polytrauma patient. The initial CT scan did not suggest biliary injury. However, intraoperatively, bile-stained fluid in the subhepatic space raised suspicion of bile duct injury. This was confirmed postoperatively in the development of a biliary fistula after the primary laparotomy. The patient was treated by endoscopic biliary stenting with complete resolution of the fistula.

## Introduction

Disruption of extrahepatic bile ducts from blunt trauma has been rarely reported [1,2]. Small tears may present as subsequent leaks leading to fistula or stricture [3]. An intraoperative cholangiogram is useful in detection and localization of these injuries [3]. A high level of suspicion is essential if the diagnosis is to be made on initial exploration [1,3]. Jaundice, nausea, vomiting, abdominal distension and pain are common symptoms of a delayed diagnosis [1,3]. A stricture or bile leak from a direct injury or an ischemic insult from injury leading to devascularization of the extrahepatic biliary tree generates these symptoms [3].

## Case presentation

A 25-year-old male came with a history of a fall from a height of approximately 30 feet from a building under construction. He sustained multiple injuries to the head, chest, abdomen, back, and extremities and presented to the hospital within a half-hour of injury. At arrival, his vital signs were: blood pressure 102/60 mm Hg, heart rate 110 beats/min, respiratory rate 40 breaths/min, oxygen saturation 80% at room air, and Glasgow coma score (GCS) 12/15 (E4 V3 M5). The airway was clear. Chest examination showed bilateral diffuse crepitations with a positive chest compression test. Abdominal examination showed mild distension and tenderness without guarding. Bowel sounds were heard. FAST (facial drooping, arm weakness, speech difficulties, time) was positive. A diagnosis of polytrauma with head, chest, and abdominal injuries was made and resuscitation started as per ATLS (advanced trauma life support) protocols. The results of the laboratory investigations performed are shown in Table 1.

**Table 1:** Laboratory investigation results AST: Aspartate aminotransferase ; ALT: Alanine transaminase

Laboratory investigations	Values found	Normal values
WBC	14.5X10^9^/L	11.0 × 10^9^/L
AST	368 U/L	10-40 U/L
ALT	358 U/L	7- 56 U/L
Hemoglobin	12.4 g/dL	12-18 g/dL
Serum bilirubin	0.2 mg/dL	0.2-1 mg/dL
Blood urea	10 mg/dL	6-24 mg/dL

A whole-body computed tomography scan was performed after initial stabilization. Grade 3 injuries of the right lobe of the liver involving segments V, VI, and VII; grade III splenic injury involving the lower pole; and grade III right kidney injury with no contrast extravasation at any site were detected on CT. Contusions were present in both lungs, with minimal pneumothorax on the left side. Multiple rib fractures were noted on the left side. Right clavicle and bilateral scapula Fractures were present. Parietal contusion was noted in the brain CT. Spine CT showed a fracture of Transverse processes of L1, L3, and L4.

Non-operative management (NOM) of the injuries was continued in the ICU with careful monitoring. Two days after admission he developed features of peritonitis along with a fall in the hemoglobin level to 9 g/dL. Emergency abdominal sonography showed free fluid which was significantly more compared to the previous scan; guided aspiration revealed blood-stained fluid and microscopic examination of the same showed multiple pus cells and gram-negative bacteria. In view of suspected peritonitis and possible bowel injury or intraabdominal bleeding, an emergency laparotomy was performed. Splenectomy was done since active bleeding from the lower pole of the spleen was discovered. There were no gastric, duodenal, gallbladder, or small/large bowel injuries. There was no active bleeding from the liver. Bile staining was seen in the area of the portahepatis but the exact site of the bile leak could not be identified. This can be prevented in the future by doing a cholangiogram which was not done in this case, however, it could definitely help to identify the leak. Splenectomy and peritoneal lavage was followed by placement of subhepatic, left subdiaphragmatic and pelvic drains. The patient remained hemodynamically stable during surgery. Immediate postoperative recovery was uneventful.

**Figure 1:**
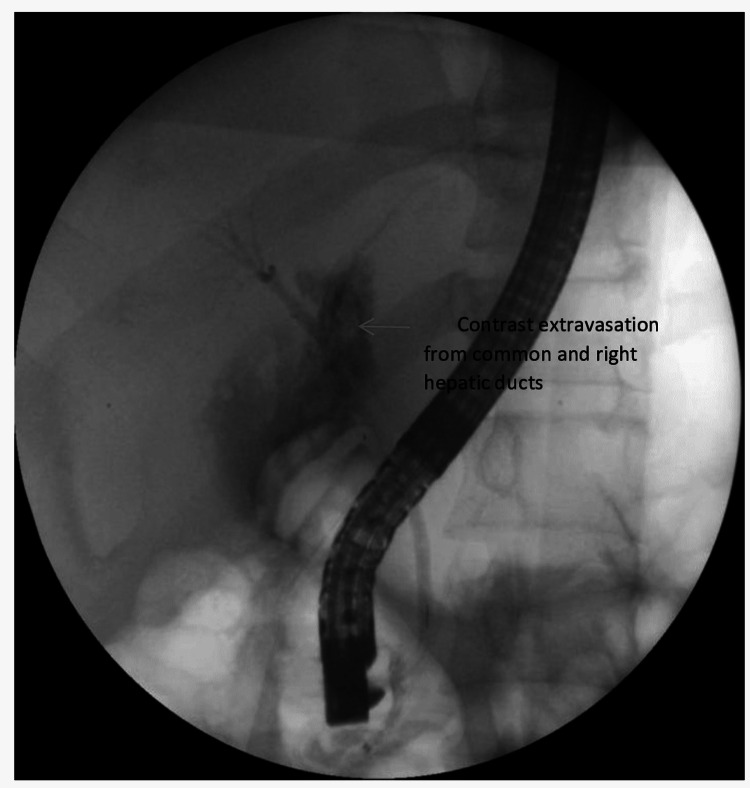
Contrast extravasation suggestive of complete disruption of common hepatic and right hepatic ducts

The patient developed a bile leak from postoperative day two through the subhepatic drain (Figure 1), with an output of 350 cc of bile per day. The patient remained hemodynamically stable with no signs of peritonitis or ileus. Additionally, on postoperative day two, he developed bilateral pleural effusion, necessitating bilateral tube thoracostomy.

**Figure 2:**
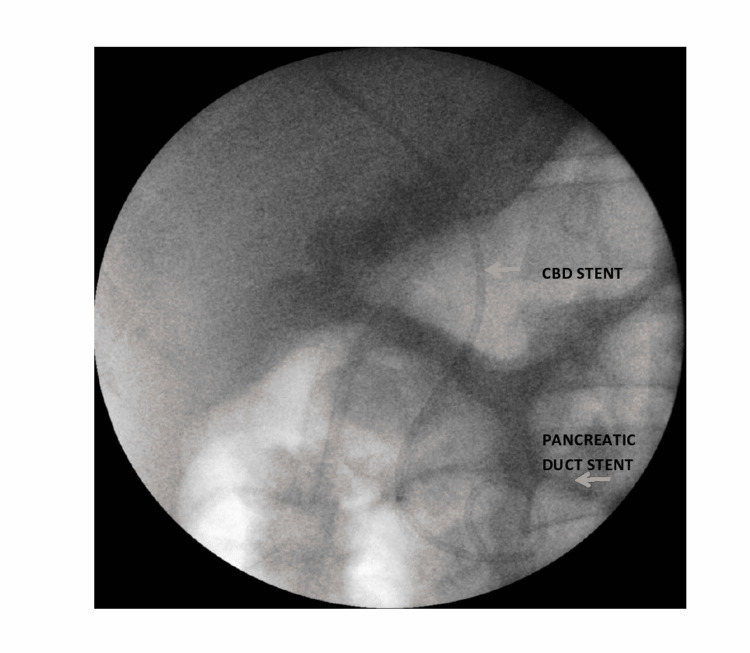
Post CBD stenting CBD: Common bile duct

Endoscopic retrograde cholangiopancreatography (ERCP) was performed on postoperative day three and showed a normal caliber common bile duct (CBD) with partial injury of the common hepatic duct. A 7F stent (Advin Healthcare, Gujarat, India) was endoscopically placed within the common hepatic and right hepatic ducts in addition to pancreatic duct stenting (Figure 2). The bile output decreased over the next seven to 10 days. 

## Discussion

An ERCP would ascertain the extent of any injuries and enable inserting stents (with sphincterotomy) around strictures or small biliary leaks, enabling these injuries to heal [1,4,5]. The upper edge of the pancreas, the hepatic duct bifurcation, and the origin of the left hepatic duct are the common areas of injury to the extrahepatic bile ducts after blunt abdominal trauma [4]. These injuries could be explained by a combination of mechanical factors such as shearing force as the liver moves cephalad, the relative rigidity of the bile ducts, rapid emptying of the gall bladder with increased internal pressure, and direct force to the duct. The increased length, elasticity, and tortuosity of the hepatic artery or portal vein explain the absence of injury to these structures [1-4].

In our patient, the diagnosis was made three days after primary laparotomy. The patient sustained a partial common hepatic duct injury with the American Association for the Surgery of Trauma (AAST) organ injury scale corresponding to Grade IV hepatic duct injuries. Options for management of these injuries involve primary closure and T-tube drainage or Roux-en-Y hepatodochojejunostomy [1-5]. End-to-end anastomosis with T-tube stent placement has been done with a complication of stricture after primary repair [2-4].

In case of delayed diagnosis, extreme inflammation or if the ends can't be approximated without tension, a biliary-enteric anastomosis is performed [2]. Since direct duct re-anastomosis has a high rate of stricture and fistula formation and biliary-enteric diversion has a low rate of these problems, the latter is recommended in all complete transections of the extrahepatic biliary tract [2]. The type and degree of ductal and hepatic damage determine the choice of treatment for hepatic duct injury.

Our patient developed repeated residual collections after stenting which was drained by percutaneous drain placement under image guidance. Studies show that ERCP with sphincterotomy and endobiliary stent placement is useful in the treatment of individuals with significant bile duct injury following trauma even if non-biliary surgical procedures have been conducted earlier [2,4,5]. ERCP stent placement resulted in complete resolution of the fistula in our patient.

Similar results were found in the research paper done by Dawson et al. where he mentions that injuries to the portal triad contents are very rare yet possible and almost never found pre-operatively which leads to significant mortality mainly due to uncontrollable hemorrhaging [6]. 

However, there are some cases where early recognition of the vague symptoms presented by the patients has been dealt with promptly and led to low morbidity and mortality. This has been portrayed by Busuttil et al. in their paper on the management of blunt and penetrating injuries to the porta hepatis [7]. The reason for the late presentation of these injuries could be either because the intraperitoneal bile fluid is sterile which makes it less damaging initially or it gets collected in large amount retroperitoneum which mask the abdominal pathology [8]. This was further worked upon by Skow and Longmire [9] who suggested that a small tear in the extrahepatic bile duct would initiate the inflammatory chain reaction which eventually would form a stricture. Longmire then suggested that the best way to treat extrahepatic injuries would be to do a duct-enteric anastomosis [10]. 

## Conclusions

Although the extrahepatic biliary injury is a rare phenomenon, its complications can be easily treated and in our patient ERCP completely resolved the patient's problems. Its injuries are generally found during surgeries. certain factors like limited imaging modalities, multiple injuries, and the late recognition of the underlying problem can pose serious implications to the prognosis of the disease. Therefore, the treatment options remain limited and largely depend on the status of the patient health. This can be improved by early imaging based on clinical suspicion and early intervention to reduce morbidity. Also, we believe that a T-tube stent should not be used in the case of a small-caliber duct. 
